# Comparative Analysis of Codon Bias in the Chloroplast Genomes of Theaceae Species

**DOI:** 10.3389/fgene.2022.824610

**Published:** 2022-03-10

**Authors:** Zhanjun Wang, Qianwen Cai, Yue Wang, Minhui Li, Chenchen Wang, Zhaoxia Wang, Chunyan Jiao, Congcong Xu, Hongyan Wang, Zhaoliang Zhang

**Affiliations:** ^1^ College of Life Sciences, Hefei Normal University, Hefei, China; ^2^ State Key Laboratory of Tea Plant Biology and Utilization, Anhui Agricultural University, Hefei, China

**Keywords:** Theaceae species, chloroplast genome, codon usage bias, expression analysis, cluster analysis

## Abstract

Theaceae species are dicotyledonous angiosperms with extremely high ornamental and economic value. The chloroplast genome is traditionally used to study species evolution, expression of chloroplast genes and chloroplast transformation. Codon usage bias (CUB) analysis is beneficial for investigations of evolutionary relationships and can be used to improve gene expression efficiency in genetic transformation research. However, there are relatively few systematic studies of the CUB in the chloroplast genomes of Theaceae species. In this study, CUB and nucleotide compositions parameters were determined by the scripts written in the Perl language, CodonW 1.4.2, CU.Win2000, RStudio and SPSS 23.0. The chloroplast genome data of 40 Theaceae species were obtained to analyse the codon usage (CU) characteristics of the coding regions and the influence of the source of variation on CUB. To explore the relationship between the CUB and gene expression levels in these 40 Theaceae plastomes, the synonymous codon usage order (SCUO) and measure independent of length and composition (MILC) values were determined. Finally, phylogenetic analysis revealed the genetic evolutionary relationships among these Theaceae species. Our results showed that based on the chloroplast genomes of these 40 Theaceae species, the CUB was for codons containing A/T bases and those that ended with A/T bases. Moreover, there was great commonality in the CUB of the Theaceae species according to comparative analysis of relative synonymous codon usage (RSCU) and relative frequency of synonymous codon (RFSC): these species had 29 identical codons with bias (RSCU > 1), and there were 19 identical high-frequency codons. The CUB of Theaceae species is mainly affected by natural selection. The SCUO value of the 40 Theaceae species was 0.23 or 0.24, and the chloroplast gene expression level was moderate, according to MILC values. Additionally, we observed a positive correlation between the SCUO and MILC values, which indicated that CUB might affect gene expression. Furthermore, the phylogenetic analysis showed that the evolutionary relationships in these 40 Theaceae species were relatively conserved. A systematic study on the CUB and expression of Theaceae species provides further evidence for their evolution and phylogeny.

## Introduction

Theaceae, a characteristic family of subtropical evergreen broad-leaved forests, is widely distributed in subtropical regions (Zhang et al., 2021). Different *Camellia* (*Camellia sinensis* L. Kuntze.) varieties are popular in many countries around the world, including India, Vietnam and Japan (Teixeira and Sousa, 2021; Wang et al., 2021). *Camellia* originated in Southeast Asia, and 80% of the species of this genus come from China. Theaceae species have high ornamental value and high economic value, such as medicine, food and drink (Yuan et al., 2021). Tea is water soaked with *Camellia* leaves, known as “oriental magic water”, which is one of three most famous drinks in the world (Balentine et al., 1997). In addition, the seeds of *Camellia* are rich in oil that can be extracted and used for various purposes, such as edible and industrial oils.

The chloroplast genome usually consists of double-stranded closed circular DNA molecules, but the chloroplast DNA of a few plants is linear. The chloroplast genome is relatively small, generally ranging from 107 to 218 kb in length, accounting for approximately 10%–20% of total plant DNA (Yao et al., 2015). The chloroplast not only has a relatively stable evolutionary rate but also has a relatively slow rate of nucleotide substitution. Thus, the investigation of the chloroplast genome is of great significance for revealing the evolutionary rules, genetic relationships, expression of chloroplast genes and chloroplast transformation among plants (Olmstead and Palmer, 1994).

Codons are the link between amino acids, proteins, and genetic materials in organisms. Meanwhile, codons play an irreplaceable role in transmitting genetic information. There are 64 codons that collectively code for 20 amino acids. Except for methionine (Met, coded by AUG) and tryptophan (Trp, coded by UGG), which have unique codons, the remaining amino acids generally have 2–6 corresponding codons; this phenomenon is known as codon degeneracy (Ikemura, 1985). Codons coding the same amino acid are called synonymous codons, and their frequencies of use in the translation process are different. This phenomenon of unbalanced codon use is called codon usage bias (CUB; Sharp and Li., 1987; Wang et al., 2020). The study of CUB is beneficial for exploring genetic evolution, understanding gene expression characteristics and providing guidance for molecular breeding.

In recent years, the rapid development of high-throughput sequencing technology has led to increasingly enriched chloroplast genome databases, laying a solid data foundation for plant genome structure and phylogenetic analyses. Thus, the chloroplast genome has been studied more extensively and in-depth. Moreover, related research on CUB in the chloroplast genome has been carried out in a variety of plants, including *Amaranthus hybridus* (Chaney et al., 2016), *Camellia oleifera* (Zhang et al., 2019), *Medicago truncatula* (Guo et al., 2020), *Nicotiana tabacum* (Arévalo-Gallegos et al., 2020); meanwhile, Magnoliaceae (Chang et al., 2006), Bambusoideae (Zhang et al., 2011), Euphorbiaceae plants (Wang et al., 2020) and *Oryza* species (Chakraborty et al., 2020). There are numerous reports on the chloroplast genome of the Theaceae species, but relatively rare on its CUB.

From the perspective of the coding sequence, we systematically analysed the codon usage (CU) characteristics of the chloroplast genomes of Theaceae species, the source of variation, the high-frequency (HF) codons, and the relationship between the CUB and gene expression levels based on measures independent of length and composition (MILC) and synonymous codon usage order (SCUO) parameters, and phylogenetic analysis was used to investigate the genetic relationships between the plants. This study revealed the relationships between the CUB and gene expression frequency, and provided a reference direction for further genetic evolution research and transgenic research in these 40 Theaceae species.

## Materials and Methods

### Source of Material

The coding regions (coding sequences, CDSs) of the chloroplast genomes of 40 Theaceae species, namely, 33 species of *Camellia*, one species of *Hartia* (*Hartia laotica*) and six species of *Stewartia*, were downloaded and collected from the National Center for Biotechnology Information (NCBI) database (https://www.ncbi.nlm.nih.gov/) on May 17, 2021. Detailed information on the chloroplast genomes of the 40 Theaceae species is provided in Table 1. To ensure the accuracy of the experimental results, the scripts written in the Perl language (Wang et al., 2020) were used to process the original CDSs of the 40 chloroplast genomes, and the selected sequences were required to meet the following conditions: 1) the CDS is composed of only A, T, G, and C bases; 2) the sequence length is a multiple of three; 3) the sequence starts with the start codon ATG and ends with the stop codon TAA, TAG or TGA; 4) there is no stop codon in the middle of the sequence (means to exclude prematurely terminated coding sequences); 5) the length of the CDS is ≥ 300 bp (He et al., 2016; Li et al., 2016; Wang et al., 2020).

**TABLE 1:** Chloroplast genome information of 40 Theaceae species.

Numbering	Species	Accession no	CDS number (before processing)	CDS number (after processing)
A	*Camellia amplexicaulis*	NC_051559.1/MT317095.1	87	58
B	*Camellia anlungensis*	NC_050354.1/MN756594.1	87	57
C	*Camellia azalea*	NC_035574.1/KY856741.1	87	56
D	*Camellia brevistyla*	NC_052752.1/MN640791.1	81	48
E	*Camellia chekiangoleosa*	NC_037472.1/MG431968.1	88	55
F	*Camellia crapnelliana*	NC_024541.1/KF753632.1	89	58
G	*Camellia cuspidata*	NC_022459.1/KF156833.1	89	58
H	*Camellia danzaiensis*	NC_022460.1/KF156834.1	87	58
I	*Camellia fascicularis*	NC_053896.1/MW026668.1	88	58
J	*Camellia fraterna*	NC_050388.1/MT663342.1	87	57
K	*Camellia gauchowensis*	NC_053541.1/MT449927.1	80	47
L	*Camellia grandibracteata*	NC_024659.1/KJ806274.1	87	56
M	*Camellia granthamiana*	NC_038181.1/MG782842.1	89	58
N	*Camellia gymnogyna*	NC_039626.1/MH394403.1	85	57
O	*Camellia impressinervis*	NC_022461.1/KF156835.1	89	58
P	*Camellia japonica S288C*	NC_036830.1/MF850254.1	89	57
Q	*Camellia kissii*	NC_053915.1/MN635793.1	88	53
R	*Camellia leptophylla*	NC_024660.1/KJ806275.1	87	56
S	*Camellia nitidissima*	NC_039645.1/MH382827.1	87	57
T	*Camellia perpetua*	NC_054364.1/MW186718.1	86	45
U	*Camellia petelotii*	NC_024661.1/KJ806276.1	87	56
V	*Camellia pitardii*	NC_022462.1/KF156837.1	89	58
W	*Camellia ptilophylla*	NC_038198.1/MG797642.1	90	57
X	*Camellia pubicosta*	NC_024662.1/KJ806277.1	87	56
Y	*Camellia pubipetala*	NC_054365.1/MW186719.1	87	57
Z	*Camellia renshanxiangiae*	NC_041672.1/MH253889.1	88	55
AA	*Camellia reticulata*	NC_024663.1/KJ806278.1	88	56
AB	*Camellia rhytidophylla*	NC_050389.1/MT663343.1	87	57
AC	*Camellia sasanqua*	NC_041473.1/MH782189.1	85	57
AD	*Camellia sinensis* var*. sinensis*	Pltd:NC_020019.1/	87	57
AE	*Camellia taliensis*	NC_022264.1/KF156839.1	89	58
AF	*Camellia yuhsienensis*	NC_053622.1/MT665973.1	82	49
AG	*Camellia yunnanensis*	NC_022463.1/KF156838.1	89	58
AH	*Hartia laotica*	NC_041509.1/MH782185.1	86	57
AI	*Stewartia micrantha*	NC_041471.1/MH782186.1	86	57
AJ	*Stewartia monadelpha*	NC_041468.1/MH782174.1	86	57
AK	*Stewartia obovata*	NC_041472.1/MH782187.1	86	57
AL	*Stewartia serrata*	NC_041467.1/MH753079.1	86	57
AM	*Stewartia sinii*	NC_041470.1/MH782182.1	86	57
AN	*Stewartia villosa*	NC_041469.1/MH782180.1	86	57

### Coding Usage Index

GC1, GC2, and GC3 respectively refer to the GC content of the first, second, and third base positions, respectively, of the chloroplast codons in the 40 Theaceae species. The GC1, GC2, and GC3 contents and the average GC content among the three sites of the 40 Theaceae species were calculated by using the scripts written in the Perl language (Wang et al., 2020). The CU index of the chloroplast CDSs was analysed by the CodonW 1.4.2 program, including T3s, C3s, A3s, and G3s, representing the frequency of T/C/A/G usage at the third base of the codon. The GC content was calculated as a percentage of the total base content, and the RSCU was determined.

### Analysis of Relative Synonymous Codon Usage (RSCU), Relative Frequency of Synonymous Codon (RFSC) and High-Frequency (HF)

RSCU is a statistical index used to weigh the relative frequency of each synonymous codon, which describes the ratio of the actual observed usage frequency of a certain codon to the expected usage frequency of the codon. Codons with an RSCU value > 1 have a positive bias, as they are used more frequently than any other synonymous codons; while an RSCU value < 1 represents negative bias, indicating that these codons are used less frequently than any other synonymous codons; an RSCU value = 1 indicates no bias, which means that synonymous codons are used equally and that CU is random (Sharp and Li, 1986). For the calculation formula of RSCU, please refer to the following formula (Eq. 1) by Sharp and Li. (1986):

$$RSCU_{ij}=\frac{x_{ij}}{\sum_{j}^{n_{i}}x_{ij}}n_{i}$$ (1)

A Toolkit for Biologists integrating various biological data-handling tools (TBtools) software was used to create a heatmap of the average RSCU values (Chen et al., 2020).

RFSC refers to the ratio of the number of codons observed in the plants to the total number of synonymous codons. The calculation formula for the frequency of use under the influence of gene length and amino acid abundance is detailed in the study of Zhou et al. (2007a) and Zhou et al. (2007b). The RFSC value can be calculated according to the actual number of codons using the CodonW 1.4.2 program (Sharp and Li, 1986). RFSC can be calculated using the following Eq. 2 (Sharp and Li, 1986):

$$RFSC=\frac{x_{ij}}{\sum_{j}^{n_{i}}x_{ij}}$$ (2)

When the RFSC value of a codon is greater than 60% or more than 1.5 times the average frequency of its synonymous codons, this codon is called an HF codon (Zhou et al., 2007a; Zhou et al., 2007b).

### Source Analysis of Codon Variation

Correspondence analysis (COA) multivariate statistical methods are often used to study the variance and gene distribution of RSCU, and each CDS was represented as a 59-dimensional vector (excluding the codon ATG encoding Met, the codon TGG encoding Trp, and the three stop codons TAA, TAG, and TGA) (Sharp and Li, 1986). The COA in CodonW 1.4.2 is generally used to study the CU variation in the genome. To reduce the influence of amino acid composition on codon utilization, each dimension corresponds to the RSCU value of each relevant codon, eliminating the influence of the stop codons TAA, TAG, and TGA.

The effective number of codons (ENc) evaluates codon use bias at the genomic level; the lower the ENc value is, the more significant the codon use preference is (Wright, 1990; Zhang et al., 2020).

$$ENc=2+S+\frac{29}{S^{2}+{\left(1-S\right)}^{2}}$$ (3)

In the ENc-GC3s plot, GC3s and ENc values were used as horizontal and vertical coordinates, respectively, to analyse the relationship between the base composition and CUB (Wang and Hickey, 2007).

Parity rule 2 (PR2) plot analysis is widely used to explain whether there is an impact on nucleotide composition due to mutation pressure and selection pressure in DNA double strands, which includes G3/(G3+C3) and A3/(A3+T3) as the horizontal and vertical coordinates, respectively. The positions of A = T and G = C (PR2) are the centres, and the coordinates are (0.5, 0.5; Meyer, 2021). The centre position of the graph indicates that there is no deviation of mutation and selectivity (replacement rate) between two complementary DNA strands. When the proportions of G and C (or A and T) are similar, the gene CUB is affected only by mutation pressure; if the composition ratio is too large, it indicates that the CUB originates from natural selection and other factors (Sueoka, 1999).

Neutral analysis is a method for quantitatively analyzing the influence of directed mutation pressure and natural selection on CUB. We examined the neutral plot and calculated the GC12 and GC3 values of the genome using the scripts written in the Perl language (Wang et al., 2020). When the slope of the regression curve goes to 0 and there is no significant correlation between GC12 and GC3, it is fully influenced by natural selection. In contrast, when the slope is close to or equal to 1 and the correlation is significant, mutation pressure is speculated to have a major effect on genes (Sueoka, 1988).

### SCUO Analysis

To better illustrate the relationship between the CUB and gene expression, we used the CU.Win2000 program to calculate the SCUO of the chloroplast sequences of the 40 Theaceae species (Wan et al., 2006). The SCUO value represents the CUB of synonymous codons relative to the whole sequence. SCUO values range between 0 and 1. The larger the value is, the stronger the CUB is. The calculation formula is explained in detail in the study by Wan et al. (2006).

### MILC Analysis

MILC is a measure of the gene expression level used to quantify the difference in CU between a gene and some expected distribution of codons. The codon distribution can either be calculated from the background nucleotide composition or derived from a single gene or a gene group (Supek and Vlahovicek, 2005). For each chloroplast genome sequence, CU analysis in R uses the package coRdon 1.13.0. First, we need to count occurrences of each codon in each sequence. The default parameter values are set during the calculation, and the MILC value of each sequence in the set is calculated for the average CU deviation of the entire sample (https://bioconductor.org/packages/devel/bioc/vignettes/coRdon/). A high MILC value indicates a high gene expression standard, and a low MILC value indicates a low gene expression standard (Dorić and Bilela, 2014).

### Phylogenetic Analysis of the 40 Theaceae Species

Two codons (AUG and UGG) and three stop codons (UAA, UAG, and UGA) encoding only one amino acid were removed from the chloroplast genomes of the 40 Theaceae species. The RSCU values of the remaining 59 synonymous codons were used as variables to calculate the distance between genes, namely, the squared Euclidian distance (Hou et al., 2016). Then, to explore the relationships between the CUB of the Theaceae species and their phylogenetic evolution in association with species relatedness, the intergroup linkage method in the SPSS 23.0 software (Pallant, 2013) was used to draw a cluster diagram for cluster analysis. The chloroplast genome sequences of 40 Theaceae species were aligned using the Muitiple Sequence Alignment Program (MAFFT v.7.4.0) (Katoh and Standley, 2013). The Maximum Likelihood phylogenetic tree of 40 Theaceae species was constructed with RAxML v.8.2.12 (Stamatakis, 2014) with 1000 bootstrap replicates, referring to the method of Liu et al. (2021). Other parameters were set by default in the software.

## Results

### Nucleotide Composition Analysis

The chloroplast coding genes of the 40 Theaceae species had very similar base compositions in the whole coding region (Table 2). The GC1, GC2 and GC3 contents were 35.84%-39.61%, 35.95%-39.88% and 35.61%-39.65%, respectively, and the mean values of the three were 37.14%-38.29%, which indicates an overall preference for codons containing A and T and ending in A/T. Therefore, from the perspective of base composition, the CUB of the chloroplast genomes of these 40 Theaceae species is extremely similar.

**TABLE 2:** Summary of the SCUO, MILC, GC, GC1, GC2, and GC3 values of the chloroplast genomes of 40 Theaceae species.

Species	SCUO	MILC	GC1%	GC2%	GC3%	GC%
*Camellia amplexicaulis*	0.23	0.55	37.48	37.93	37.23	37.55
*Camellia anlungensis*	0.24	0.56	38.16	36.80	37.72	37.56
*Camellia azalea*	0.24	0.55	38.45	37.43	36.80	37.56
*Camellia brevistyla*	0.24	0.56	37.79	36.42	37.20	37.14
*Camellia chekiangoleosa*	0.24	0.56	37.47	36.59	38.22	37.42
*Camellia crapnelliana*	0.24	0.55	37.31	37.41	37.93	37.55
*Camellia cuspidata*	0.24	0.55	36.89	39.04	36.76	37.57
*Camellia danzaiensis*	0.24	0.55	35.84	37.26	39.60	37.57
*Camellia fascicularis*	0.23	0.55	37.16	37.77	37.71	37.55
*Camellia fraterna*	0.24	0.55	37.55	38.25	36.99	37.60
*Camellia gauchowensis*	0.24	0.55	36.09	37.37	38.32	37.26
*Camellia grandibracteata*	0.24	0.56	37.30	38.76	36.65	37.57
*Camellia granthamiana*	0.23	0.55	38.69	37.21	36.75	37.55
*Camellia gymnogyna*	0.24	0.55	36.73	37.56	38.41	37.56
*Camellia impressinervis*	0.24	0.55	37.22	38.54	36.88	37.55
*Camellia japonica S288C*	0.23	0.55	38.37	36.44	37.75	37.52
*Camellia kissii*	0.23	0.55	38.23	37.20	37.29	37.57
*Camellia leptophylla*	0.24	0.55	37.58	36.66	38.50	37.58
*Camellia nitidissima*	0.24	0.55	38.02	36.41	38.19	37.54
*Camellia perpetua*	0.24	0.55	36.69	36.38	39.08	37.38
*Camellia petelotii*	0.24	0.56	37.34	36.39	38.97	37.57
*Camellia pitardii*	0.24	0.55	38.49	36.31	37.89	37.57
*Camellia ptilophylla*	0.23	0.55	37.79	38.29	36.51	37.53
*Camellia pubicosta*	0.24	0.56	37.92	35.95	38.86	37.58
*Camellia pubipetala*	0.24	0.56	38.56	37.06	36.34	37.32
*Camellia renshanxiangiae*	0.24	0.56	38.73	37.79	38.27	38.27
*Camellia reticulata*	0.23	0.55	38.92	37.20	36.53	37.55
*Camellia rhytidophylla*	0.24	0.55	36.78	39.20	36.81	37.60
*Camellia sasanqua*	0.24	0.55	38.16	37.65	38.77	38.19
*Camellia sinensis* var*. sinensis*	0.24	0.55	36.57	39.88	36.23	37.56
*Camellia taliensis*	0.24	0.55	38.54	36.72	37.45	37.57
*Camellia yuhsienensis*	0.24	0.55	38.33	37.88	35.61	37.27
*Camellia yunnanensis*	0.24	0.55	36.35	38.22	38.08	37.55
*Hartia laotica*	0.23	0.55	38.20	39.08	37.56	38.28
*Stewartia micrantha*	0.23	0.55	37.65	37.53	39.65	38.28
*Stewartia monadelpha*	0.23	0.56	39.20	36.95	38.56	38.24
*Stewartia obovata*	0.23	0.55	37.56	39.39	37.92	38.29
*Stewartia serrata*	0.23	0.55	38.18	37.37	39.17	38.24
*Stewartia sinii*	0.23	0.55	39.61	36.16	39.01	38.26
Stewartia villosa	0.23	0.55	39.30	37.68	37.89	38.29

### RSCU, RFSC Analysis and HF Codon Screening Reveal Biased Use of A/T

**FIGURE 1:**
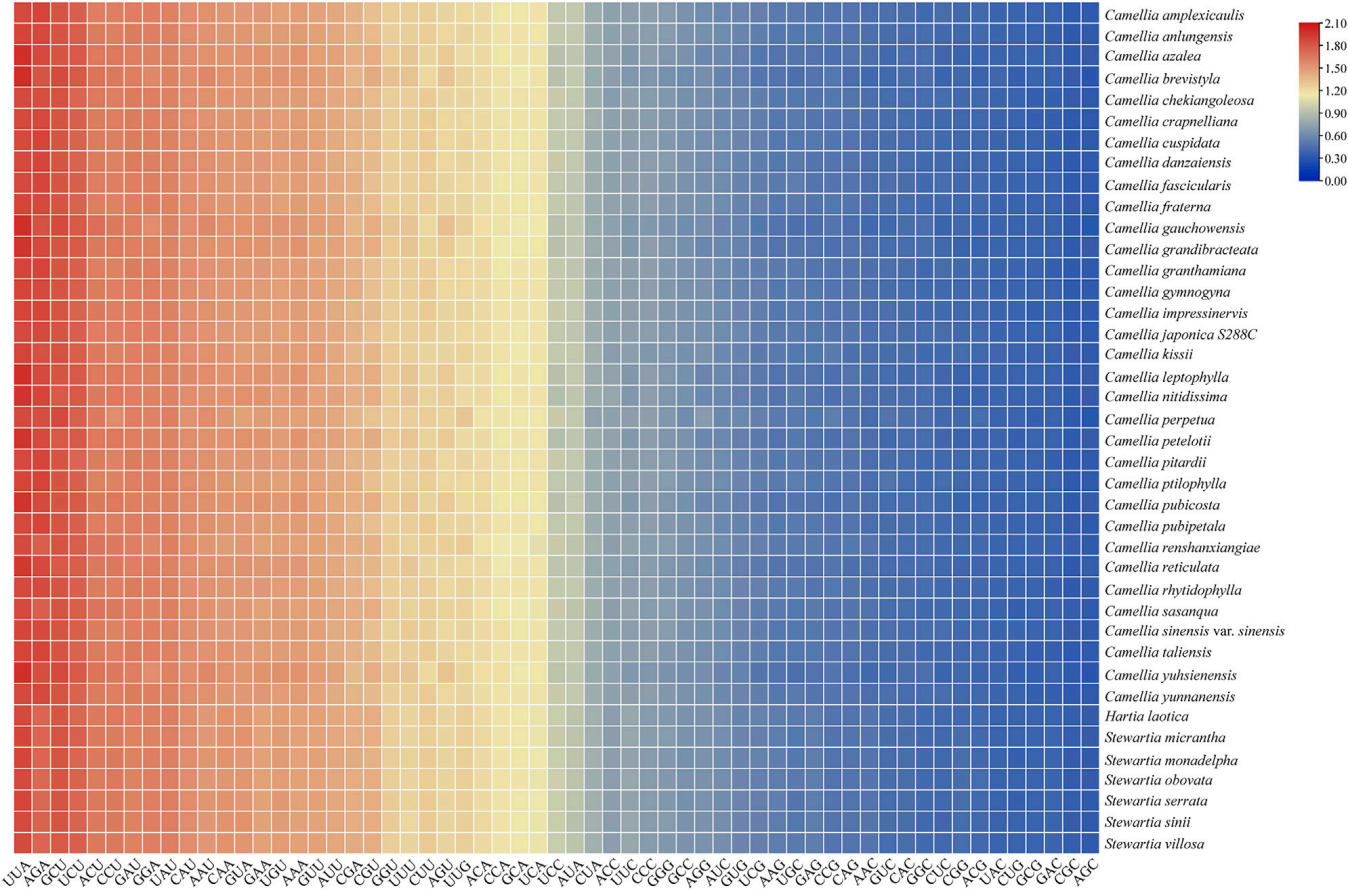
The RSCU values of the chloroplast genomes in 40 Theaceae species. A gradient from blue to red indicates that the average RSCU value of the codon is from low to high.

As shown in Figure 1, the number of preferred codons (RSCU > 1) in the 40 chloroplast genomes was 29, among which, 28 (96.55%) ended in A or U (excluding UUG, 12 ended in A and 16 ended in U), the probability of the preferred codon ending in A/T, was much higher than that ending in G/C. In all the Theaceae species, AGA, encoding arginine (Arg), was strongly preferred (RSCU > 1.7), followed by GAU, encoding aspartic acid (Asp). The RFSC value was calculated by using the actual number of codons used by the CodonW 1.4.2 program (Supplementary Table S1). In summary, the codons in the chloroplast genomes of the 40 Theaceae species all showed a preference for ending in A/U. Furthermore, in Figure 1, the colours of the 40 Theaceae species for the same codon were extremely similar, indicating that the RSCU values for a given codon in the chloroplast genomes of the Theaceae species were similar. The HF codons in the chloroplast genomes of the 40 Theaceae species are shown in Table 3. The HF codons encoding 19 amino acids (Ala, Cys, Asp, Glu, Phe, Gly, His, Ile, Lys, Leu, Asn, Pro, Gln, Arg, Ser, Thr, Val, Tyr, and TER) are highly similar, with only a few codons showing some differences (Phe, Gln, Val, TER). There were 18 common HF codons (GCU, UGU, GAU, GAA, UUU, GGA, CAU, AUU, AAA, UUA, AAU, CCU, CAA, AGA, UCU, ACU, GUA, and UAU) in the chloroplast genomes, and only the codons UUC, CAG, GUU and UAA were different.

**TABLE 3:** Screening of high-frequency codons in the chloroplast genomes of the 40 Theaceae species.

Amino acid	High-frequency codons
A(Ala)	GCU^h^
C(Cys)	UGU^h^
D(Asp)	GAU^h^
E(Glu)	GAA^h^
F(Phe)	UUU^h^ , **UUC** ^**h**^
G(Gly)	GGA^h^
H(His)	CAU^h^
I(Ile)	AUU^h^
K(Lys)	AAA^h^
L(Leu)	UUA^h^
N(Asn)	AAU^h^
P(Pro)	CCU^h^
Q(Gln)	CAA^h^ , **CAG** ^**h**^
R(Arg)	AGA^h^
S(Ser)	UCU^h^
T(Thr)	ACU^h^
V(Val)	GUA^h^ , **GUU** ^**h**^
Y(Tyr)	UAU^h^
TER	**UAA** ^**h**^

Codons in bold font are high-frequency codons with differences in the chloroplast genomes of 40 Theaceae species.

### Analysis of Codon Variation Suggests Natural Selection Dominates

**FIGURE 2:**
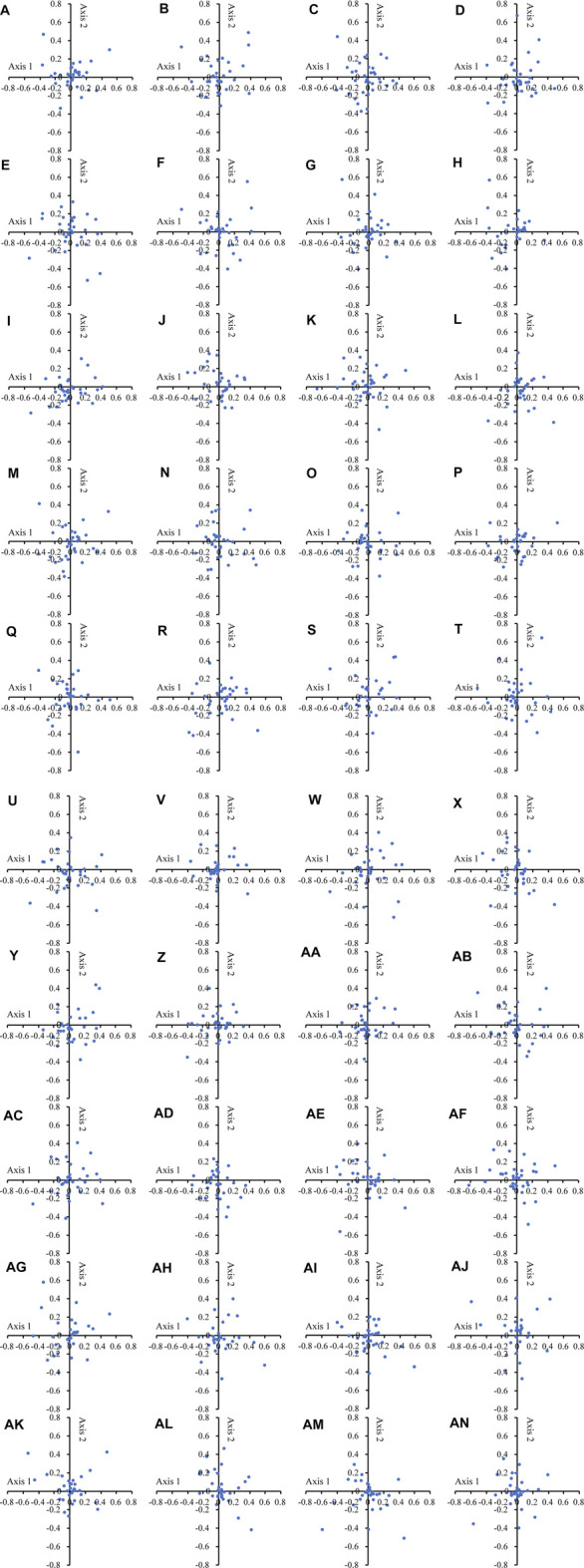
COA analysis of the chloroplast genomes in 40 Theaceae species. In all the Theaceae species, the dots of different genes are separated from each other in the figure. The numbers **(A–AN)** of the 40 species are shown in Table 1.

In the COA of the 40 Theaceae species (Figure 2), the points representing different genes were separated from each other in all plants of *Camellia*. In the chloroplast genomes of the 40 Theaceae species, axis 1 and axis 2 accounted for 9.32%-10.73% and 8.05%-9.95% of the synonymous CU variation, respectively. Genes with different GC contents are marked with different colours to reveal the influence of GC content on CUB. The GC content of all the genes in the 40 Theaceae species was less than 45%. This high consistency indicates that the sources of CU variation in different Theaceae species are extremely similar.

**FIGURE 3:**
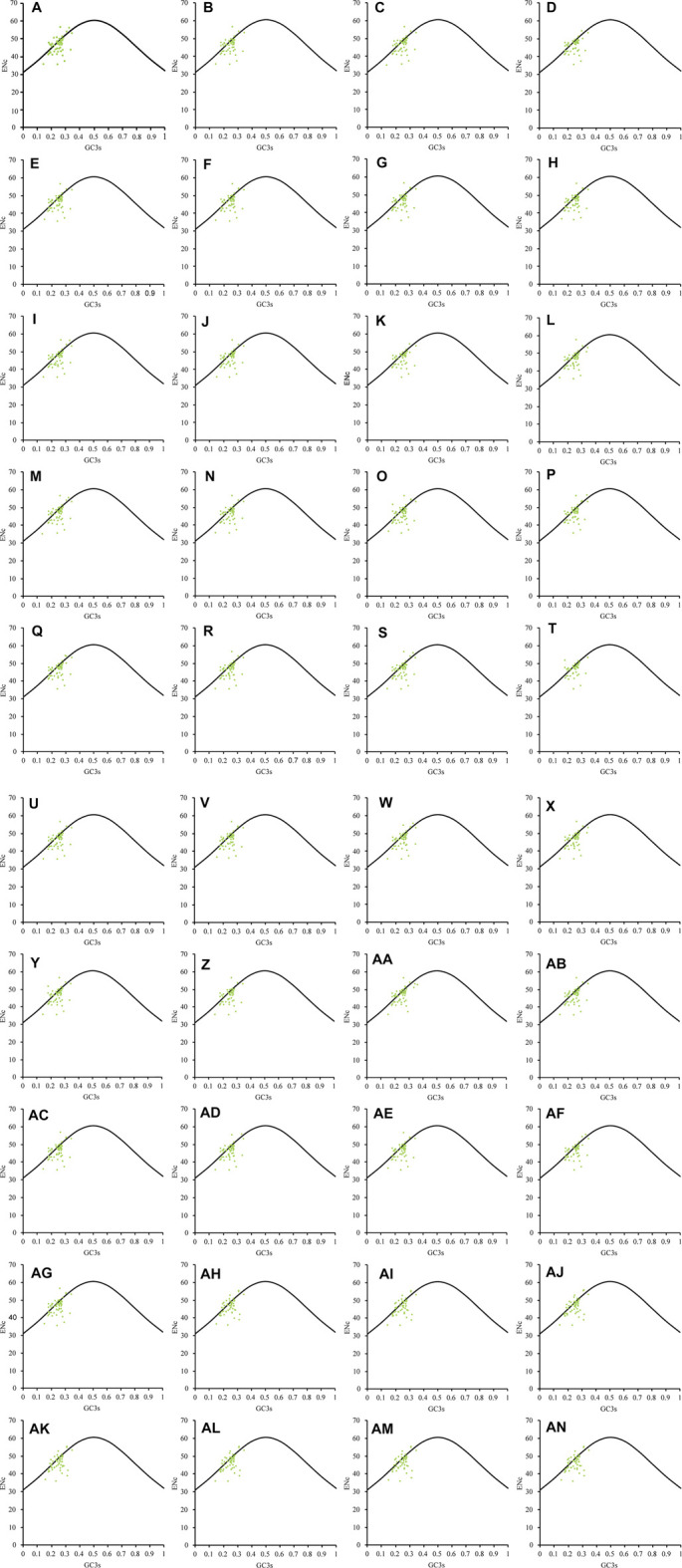
ENc-GC3s plot of the chloroplast genomes in 40 Theaceae species. The chloroplast genomes of the 40 Theaceae species are generally scattered in small clusters, and the genes are distributed on the left side of the figure. The numbers **(A–AN)** of the 40 species are shown in Table 1.

In the ENc-GC3s map of the 40 Theaceae species (Figure 3), all the genes were scattered in small clusters on the left side. Based on observation and analysis, the chloroplast genomes of the 40 Theaceae species showed scattered small clusters overall. Most of the genes were distributed far away from the standard curve and others were distributed in the appendix of the standard curve. This shows that the CUB of the chloroplast genomes is affected not only by mutation pressure but also by natural selection and other factors.

**FIGURE 4:**
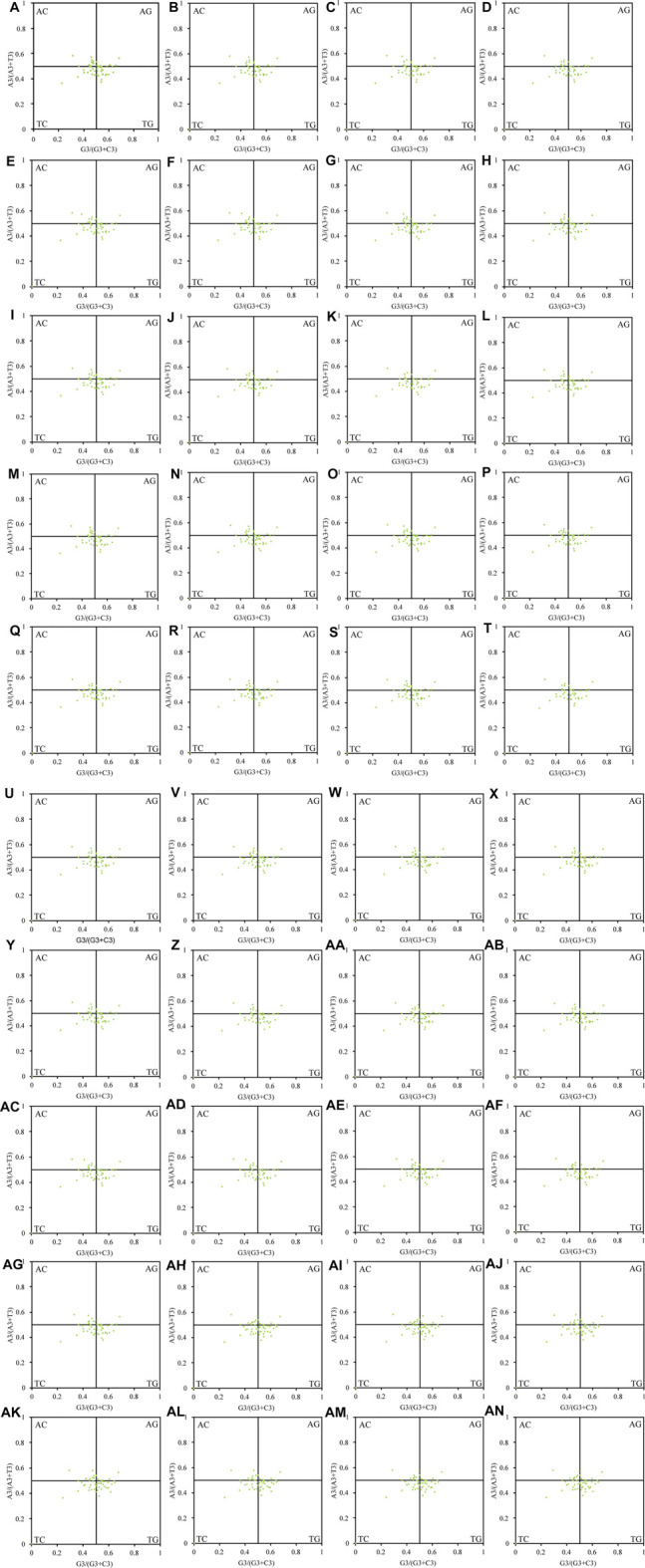
PR2 plot of the chloroplast genomes in 40 Theaceae species. Points are randomly distributed in the four quadrants, and are mainly distributed in the regions of G3/(G3+C3) > 0.5 and A3/(A3+T3) < 0.5. The numbers **(A–AN)** of the 40 species are shown in Table 1.

**FIGURE 5:**
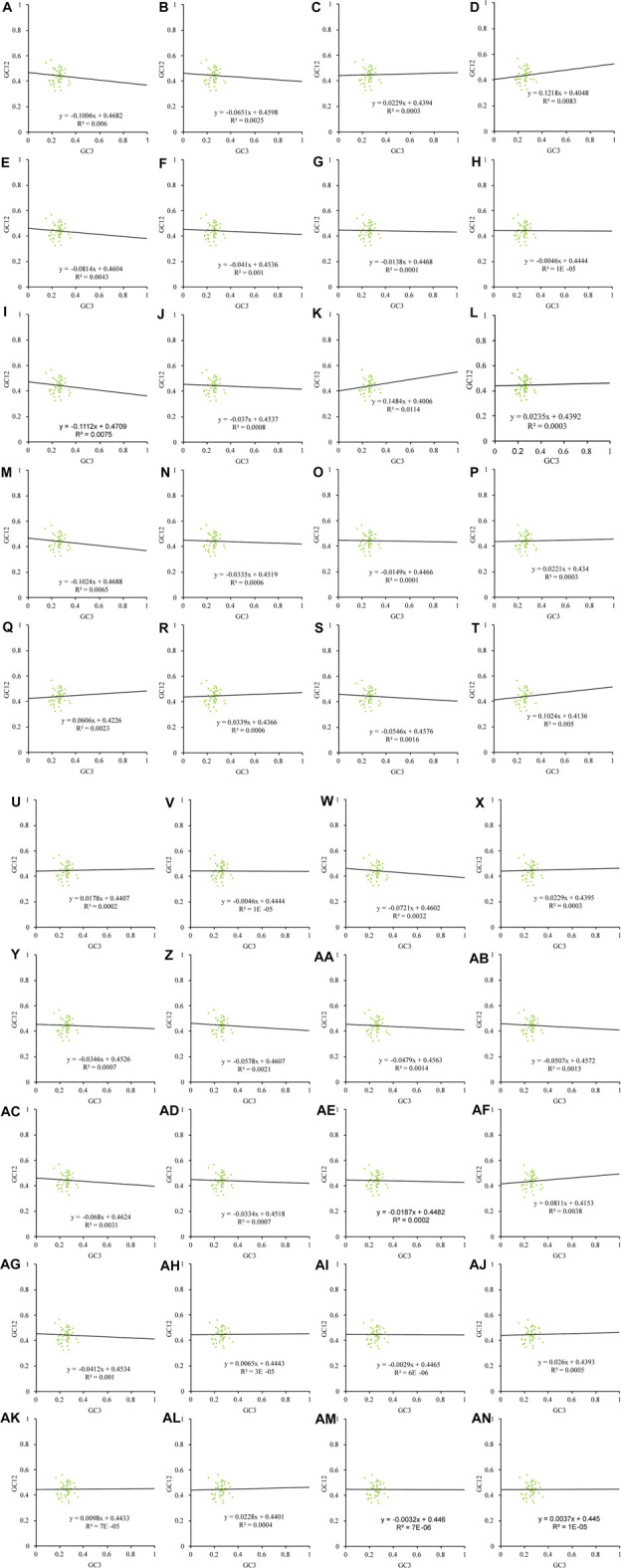
Neutral analysis of the chloroplast genomes in 40 Theaceae species. The distribution range of GC12 and GC3 is relatively narrow, GC12 is distributed between 0.32 and 0.57, GC3 is distributed between 0.14 and 0.36, most of the points are distributed in small clusters. The numbers **(A–AN)** of the 40 species are shown in Table 1.

Natural selection may affect the use of bases A and T together with G and C. In this study, we found that the chloroplast genes of the 40 Theaceae species were distributed unevenly in the four quadrants of the PR2 plot (Figure 4), and they were distributed mainly in the regions of G3/(G3+C3) > 0.5 and A3/(A3+T3) < 0.5, indicating that the use of the third codon was unbalanced. Figure 5 shows an important positive correlation between GC12 and GC3 of the chloroplast genomes (*P* ≤ 0.01), which indicates that all the Theaceae species have been subject to direct mutation pressure on chloroplast gene CU. In the neutral analysis chart, the distribution ranges of GC12 and GC3 were relatively narrow. GC12 was distributed between 0.32 and 0.57, and GC3 was distributed between 0.14 and 0.36. Most points were distributed in small clusters, indicating that both natural selection and mutation pressure affect CUB.

The neutral analysis chart can further reveal the strength of mutation pressure and natural selection. When the slope of the regression line is close to 0, natural selection plays a leading role, and when the slope of the regression line is close to 1, rapid mutation pressure is assumed to play a main role. Among six *Camellia* species, namely, *Camellia amplexicaulis*, *Camellia brevistyla*, *Camellia fascicularis*, *Camellia gauchowensis*, *Camellia granthamiana*, and *Camellia leptophylla*, the absolute value of the angle at which the regression line slopes was slightly greater than 0.1, including −0.1006, 0.1218, 0.1112, 0.1484, −0.1024 and 0.1024, respectively, indicating that the mutation pressure accounted for approximately 10% of the variation, which means that natural selection plays an important role; additionally, among the other 34 Theaceae species, natural selection had an effect on CUB as high as 90%.

### SCUO Analysis

SCUO was used as an important parameter to analyse CUB in the chloroplast genomes of the 40 Theaceae species. The average SCUO value of each chloroplast genome is shown in Table 2. The average SCUO of the chloroplast genome of 14 species of Theaceae species was 0.23, while the average SCUO of the other 26 species of Theaceae species was 0.24, both of which are lower than the median value of 0.5, which indicates that most genes in the chloroplast genome of Theaceae species have a low CUB. In summary, from the perspective of SCUO, the CUB of the chloroplast genomes of the 40 Theaceae species is very similar.

### CUB Affects Gene Expression

The MILC value reflects the level of gene expression. The greater the MILC number is, the higher the gene expression levels is. We calculated the MILC value of chloroplast genes of the 40 Theaceae species (Supplementary Table S2), and the results showed that 31 Theaceae species had a MILC value of 0.55, and the other 9 species had a MILC value of 0.56, which revealed that the expression level of most of the chloroplast genes was moderate. Additionally, we used correlation analysis to explore the relationship between the gene expression level and the CUB. We observed a significant positive correlation between the SCUO and MILC values (Table 4), indicating that the gene expression levels may be affected by the CUB.

**TABLE 4:** Correlation between the SCUO and MILC values in the different chloroplast genomes of 40 Theaceae species.

Species	*r*	*P*
*Camellia amplexicaulis*	0.463^**^	0.000
*Camellia anlungensis*	0.461^**^	0.000
*Camellia azalea*	0.467^**^	0.000
*Camellia brevistyla*	0.398^**^	0.002
*Camellia chekiangoleosa*	0.448^**^	0.001
*Camellia crapnelliana*	0.444^**^	0.000
*Camellia cuspidata*	0.440^**^	0.001
*Camellia danzaiensis*	0.419^**^	0.001
*Camellia fascicularis*	0.455^**^	0.000
*Camellia fraterna*	0.456^**^	0.000
*Camellia gauchowensis*	0.483^**^	0.000
*Camellia grandibracteata*	0.478^**^	0.000
*Camellia granthamiana*	0.426^**^	0.001
*Camellia gymnogyna*	0.450^**^	0.000
*Camellia impressinervis*	0.458^**^	0.000
*Camellia japonica S288C*	0.459^**^	0.000
*Camellia kissii*	0.449^**^	0.000
*Camellia leptophylla*	0.418^**^	0.001
*Camellia nitidissima*	0.467^**^	0.000
*Camellia perpetua*	0.459^**^	0.001
*Camellia petelotii*	0.418^**^	0.001
*Camellia pitardii*	0.467^**^	0.000
*Camellia ptilophylla*	0.480^**^	0.001
*Camellia pubicosta*	0.490^**^	0.000
*Camellia pubipetala*	0.440^**^	0.000
*Camellia renshanxiangiae*	0.432^**^	0.000
*Camellia reticulata*	0.478^**^	0.005
*Camellia rhytidophylla*	0.398^**^	0.001
*Camellia sasanqua*	0.480^**^	0.000
*Camellia sinensis* var*. sinensis*	0.466^**^	0.001
*Camellia taliensis*	0.450^**^	0.000
*Camellia yuhsienensis*	0.443^**^	0.000
*Camellia yunnanensis*	0.451^**^	0.000
*Hartia laotica*	0.435^**^	0.000
*Stewartia micrantha*	0.416^**^	0.001
*Stewartia monadelpha*	0.440^**^	0.000
*Stewartia obovata*	0.480^**^	0.000
*Stewartia serrata*	0.461^**^	0.001
*Stewartia sinii*	0.482^**^	0.001
*Stewartia villosa*	0.383^**^	0.000

**Significant at the *P* < 0.01 level (two-tailed).

### Phylogenetic Analysis of the 40 Theaceae Species

**FIGURE 6:**
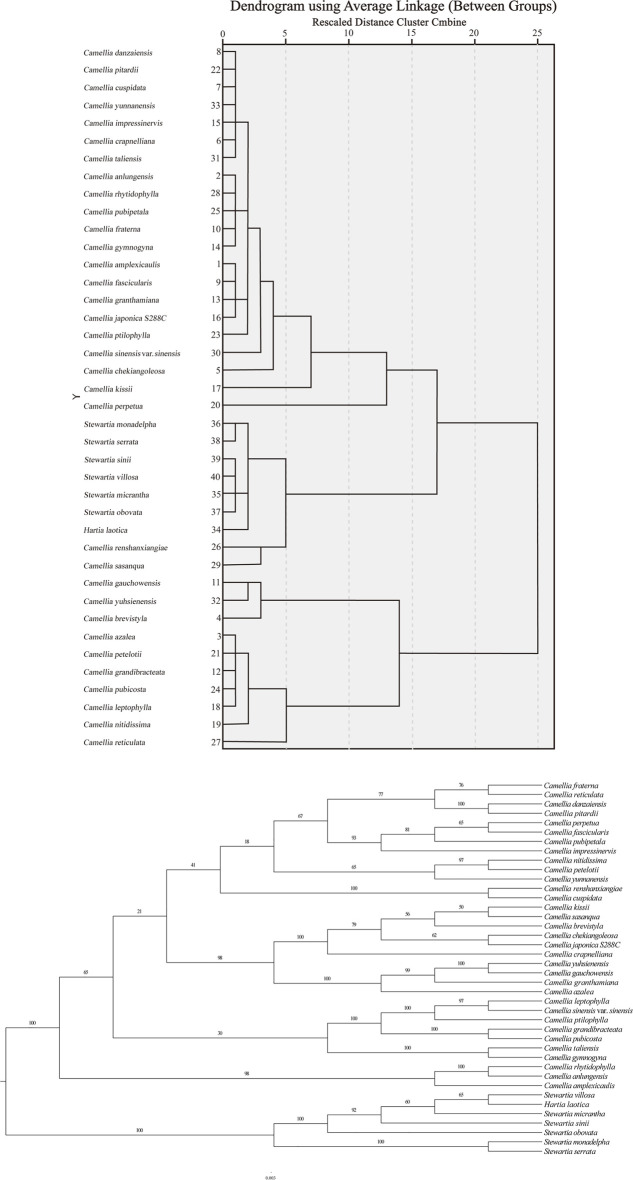
Phylogenetic analysis of the chloroplast genomes in 40 Theaceae species. The clustering results and the phylogenetic tree are divided into two branches. The topology of the phylogenetic tree is similar to the clustering results based on the codon RSCU values of the chloroplast genome to a certain extent.

The phylogenetic tree constructed based on the cluster analysis of the RSCU values and the ML analysis of the chloroplast genomes has a highly similar topology. The greater the squared Euclidean distance between plants is, the greater the difference in their CUB. According to the RSCU calculations, the squared Euclidean distance between the chloroplast genes of the 40 Theaceae species was extremely small (all less than 0.12). At the same time, the clustering results divided the plants into two major branches: the first branch contained ten species of *Camellia*, all belonging to the genus *Camellia*; the second branch contained 30 of Theaceae, including 23 species of *Camellia*, one species of *Hartia* (*Hartia laotica*) and six species of *Stewartia* species (Figure 6). On the first branch, *Camellia brevistyla*, *Camellia gauchowensis* and *Camellia yuhsienensis* formed a small group, while the remaining plants belonged to another group: *Camellia azalea*, *Camellia petelotii*, *Camellia grandibracteata*, *Camellia pubicosta*, *Camellia leptophylla*, *Camellia Nitidissima*, and *Camellia reticulata*. On the second branch, two species of *Camellia* (*Camellia monadelpha* and *Camelliaserrata*), one species of *Hartia* (*Hartia laotica*) and six species of *Stewartia* formed a small group, with the two species of *Camellia* (*Camellia renshanxiae* and *Camellia sasanqua*) forming their own small group. The other 21 species of *Camellia* belonged to another small group. The results showed that the 21 species of *Camellia* in the second clade were more closely related, while the 10 species in the first clade were more distantly related.

The results of the constructed phylogenetic tree based on the genomes are samely divided into two major branches: the first branch contains 33 species of *Camellia* and all belong to the genus *Camellia*, and the second branch contains the remaining seven species of *Camellia*, including one species of *Hartia* (*Hartia laotica*) and six species of *Stewartia*. However, the six species of *Stewartia* belong to a small branch in phylogenetic tree according to the RSCU values. Overall, the topological structure of the phylogenetic tree and the clustering results based on the RSCU values of the chloroplast genome are similar.

## Discussion

Codons are an important core element that connects amino acids, proteins and genetic materials in living organisms (Sundararajan et al., 2016). Studies of the preferred usage bias of codons provide reliable information for the study of protein expression and its corresponding functions (Wang et al., 2018). Many biological factors have been demonstrated to influence CUB, including gene length, tRNA quantity, gene expression level, chain-specific mutation bias, and GC composition (Wan et al., 2004; Meyer, 2021).

Therefore, this study systematically analysed the CUB characteristics, CUB variation sources and phylogenetic relationships of the chloroplast genomes of 40 Theaceae species. As claimed by the results of RSCU, RFSC analysis and HF codon screening, the codons tended to end with A/T, which is consistent with the assumption that “higher plants tend to use codons ending with A/T” (Campbell and Gowri, 1990; Wang et al., 2020). According to Sharp et al. (1988) proposed that “organisms with close genetic relationships have very similar codon usage bias”, the CUB in the chloroplast genomes of the 40 Theaceae species are extremely similar based on the RSCU values and comparative analysis of CU. In addition, a CUB analysis of 18 rice chloroplast genomes revealed that their CUB was low and AT base preference was strong; different rice varieties have different CU patterns that are mainly subjected to mutation pressure and natural selection (Chakraborty et al., 2020). We also analysed the preferred codons and HF codons of the 40 Theaceae species (Campbell and Gowri, 1990); specifically, there was very high similarity in the use of codons among the 40 Theaceae species. Eighteen codons were both preferred codons and HF codons: GCU, UGU, GAU, GAA, UUU, GGA, CAU, AUU, AAA, UUA, AAU, CCU, CAA, AGA, UCU, ACU, GUA, and UAU. Compared to the use of one type of codon (preferred codons or HF codons), the use of the two types (preferred codons and HF codons) may have a higher reference value for the optimization of codons in terms of heterologous expression.

Regarding the source of codon variation, our results further suggested that both mutation pressure and natural selection affect CUB in the chloroplast genomes of the 40 Theaceae species. ENc-GC3s and PR2 plot analysis showed that the CUB of the 40 Theaceae species was influenced by mutation pressure and natural selection, and the similar results were found in other tea plants (Rawal et al., 2020; Rawal et al., 2021). In addition, a natural selection study of the chloroplast genomes of 29 species of Magnoliaceae systematically compared their CU characteristics and elucidated their evolutionary relationships (Chang et al., 2006). Meanwhile, Zhang et al. (2011) get the similar conclusion on 3 species in the bamboo subfamily. Comparisons of plant chloroplast genomes have revealed the main reason for their CUB. Furthermore, the quantitative results of neutral analysis showed that the CUB of the 40 Theaceae species were mainly affected by natural selection, which was consistent with the influencing factors of CUB in the chloroplast genomes of Magnoliaceae (Chang et al., 2006) and Euphorbiaceae plant ( Wang et al., 2020). In summary, natural selection plays a leading role while mutation pressure plays a secondary role in determining the CU of chloroplast genes in 40 Theaceae species.

In our study, the calculated SCUO values were lower than 0.25, which means that most genes in the chloroplast-encoding genome of Theaceae plastomes have a low bias. This result is consistent with the result of chloroplast genes in *Oryza* species (Chakraborty et al., 2020). In *Camellia amplexicaulis*, *Camellia fascicularis*, *Camellia granthamiana*, *Camellia japonica S288C*, *Camellia kissii*, *Camellia ptilophylla*, *Camellia reticulata*, *Hartia laotica*, *Stewartia micrantha*, *Stewartia monadelpha*, *Stewartia obovata*, *Stewartia serrata*, *Stewartia sinii*, and *Stewartia villosa*, the average value of SCUO was 0.23, which reflects the low codon bias. In the results of 18 *Oryza* species studies, the SCUO values ranged from 0.24 to 0.27, indicating that the CUB was low and varied in most of the rice species. Likewise, the 40 Theaceae species had low chloroplast CUB. We regarded MILC as a measure of gene expression in our current study and observed high MILC values. The MILC value of 31 of the Theaceae species was 0.55, and the value of the other nine kinds of MILC was 0.56. In the related research on *Oryza* species, the expression level of each chloroplast gene of 18 rice species based on the MILC value was calculated to be in the range of 0.74 to 0.78, which means that most of the chloroplast genes are expressed at high levels in rice and that there are differences in gene expression levels (Chakraborty et al., 2020). The results showed that, from the perspective of MILC, there may be certain differences in gene expression between dicotyledonous plants and monocotyledonous plants of chloroplast genomes. Karl Pearson’s correlation analysis between SCUO and MILC was used to explore the effect of CUB (Sareen et al., 2018). Based on the gene expression level, we observed a significant positive between SCUO and MILC values (*P* < 0.01; Table 4), indicating that the gene expression levels may be affected by the CUB. All in all, the higher the CUB is, the higher the gene expression level is (Li et al., 2016). In related reports by Zhao et al. (2016) and Liu et al. (2020), there is a close correlation between gene expression frequency and CUB, that is, the stronger the CUB, the higher the gene expression frequency. The efficiency of gene expression can be improved by codon optimization. For example, enhancing the expression of viral genes in mammalian cell lines by complete resynthesis of the gene (Gustafsson et al., 2004). Based on the common use characteristics of the codons identified in the above analysis, the expression efficiency of some exogenous genes in the Theaceae species can be improved, and the functional genes regulating the growth and development of the Theaceae species can be targeted (Peng et al., 2021).

There are many species of plants in Theaceae, and the interspecific relationship of this economically important plant is still a very controversial issue (Vijayan et al., 2009; Yang et al., 2013). The two classification approaches proposed by Chang (1998) and Ming (2000) diverge in many respects, especially with regard to the division of subgenera, sections and species. So far, it is uncertain which system has most accurately described the phylogenetic relationships within the Theaceae family. Therefore, it is necessary to look for other evidence to reconstruct the establishment of the Theaceae classification system. The squared Euclidean distances between plants within the genera *Camellia* and *Stewartia* were smaller (≤ 0.1), indicating that the CUB of the chloroplast genes were very similar (Wang, 2007). Based on the cluster analysis map constructed by RSCU values, most species of the same genus are clustered together, indicating that closely related species tend to have the same codon bias pattern. This is consistent with the conclusion in Liu et al. (2005) research that the closer the genetic relationship between species, the more similar the CU patterns. In addition, the clustering results based on RSCU values were similar to the topological structure of the phylogenetic tree constructed based on chloroplast genome sequences. In the topology of the phylogenetic tree, *Camellia chekiangoleosa*, *Camellia crapnelliana*, *Camellia cuspidat*, *Camellia granthamiana*, *Camellia gymnogyna*, *Camellia impressinervis*, *Camellia pitardii*, *Camellia ptilophylla*, *Camellia renshanxiangiae*, *Camellia yunnanensis* belongs to the first clade, which has the same opinion with Li et al. (2021). And *Camellia pitardii* was clustered with *Camellia crapnelliana*, *Camellia cuspidate*, *Camellia impressinervis*, and *Camellia yunnanensis*, indicating that *Camellia pitardii* might belong to subgenus *Camellia*, and it means they’re very closely related, which is similar to Li et al. (2021). In addition, there are some inconsistencies between the phylogenetic tree of this study and others’ Theaceae phylogeny. Li et al. (2021) is mainly focus on the phylogeny of *Camellia, Camellia renshanxiangiae* and *Camellia sasanqua* belong to the genus *Camellia*. However, in cluster analysis based on RSCU values, the two species of *Camellia* grouped into the *Stewartia-Hartia* clade. This provides a clearer direction for our subsequent research in the further. Therefore, the clustering method based on RSCU values can be used as a supplement to the molecular taxonomy and phylogenetic studies of Theaceae species. Furthermore, based on the genetic relationships revealed by cluster analysis, it is expected that in the future, transgenic technology can be used to conduct molecular experiments on genes underlying important traits in Theaceae species in order to facilitate in-depth studies on the cultivation of Theaceae species.

## Data Availability

The original contributions presented in the study are included in the article/Supplementary Material, further inquiries can be directed to the corresponding author.
