# Supplementary material for: Comparative Analysis of Codon Bias in the Chloroplast Genomes of Theaceae Species
Source: Front Genet. 2022 Mar 10;13:824610. doi: 10.3389/fgene.2022.824610 (PMC8961065; doi:10.3389/fgene.2022.824610)
Supplement: Supplementary file 1 [file Table2.DOCX]

**Supplementary Table 2** The MILC values of each cp gene in 40 Theaceae species.

| **Species**  **MILC** | |
| --- | --- |
| *Camellia amplexicaulis* | 0.55 |
| *Camellia anlungensis* | 0.56 |
| *Camellia azalea* | 0.55 |
| *Camellia brevistyla* | 0.56 |
| *Camellia chekiangoleosa* | 0.56 |
| *Camellia crapnelliana* | 0.55 |
| *Camellia cuspidata* | 0.55 |
| *Camellia danzaiensis* | 0.55 |
| *Camellia fascicularis* | 0.55 |
| *Camellia fraterna* | 0.55 |
| *Camellia gauchowensis* | 0.55 |
| *Camellia grandibracteata* | 0.56 |
| *Camellia granthamiana* | 0.55 |
| *Camellia gymnogyna* | 0.55 |
| *Camellia impressinervis* | 0.55 |
| *Camellia japonica S288C* | 0.55 |
| *Camellia kissii* | 0.55 |
| *Camellia leptophylla* | 0.55 |
| *Camellia nitidissima* | 0.55 |
| *Camellia perpetua* | 0.55 |
| *Camellia petelotii* | 0.56 |
| *Camellia pitardii* | 0.55 |
| *Camellia ptilophylla* | 0.55 |
| *Camellia pubicosta* | 0.56 |
| *Camellia pubipetala* | 0.56 |
| *Camellia renshanxiangiae* | 0.56 |
| *Camellia reticulata* | 0.55 |
| *Camellia rhytidophylla* | 0.55 |
| *Camellia sasanqua* | 0.55 |
| *Camellia sinensis var. sinensis* | 0.55 |
| *Camellia taliensis* | 0.55 |
| *Camellia yuhsienensis* | 0.55 |
| *Camellia yunnanensis* | 0.55 |
| *Hartia laotica* | 0.55 |
| *Stewartia micrantha* | 0.55 |
| *Stewartia monadelpha* | 0.56 |
| *Stewartia obovata* | 0.55 |
| *Stewartia serrata* | 0.55 |
| *Stewartia sinii* | 0.55 |
| *Stewartia villosa* | 0.55 |
